# Intergenerational transmission of health disparities among Turkish-origin immigrants in Germany: study protocol of a multi-centric cohort study (BaBi-stress and BaBeK study)

**DOI:** 10.1186/s12884-020-2853-y

**Published:** 2020-03-12

**Authors:** Jacob Spallek, Laura Scholaske, Medlin Kurt, Denise Lindner-Matthes, Sonja Entringer

**Affiliations:** 1 Department of Public Health, Brandenburg University of Technology, Universitätsplatz 1, 01968 Senftenberg, Germany; 2 Charité – Universitätsmedizin Berlin, corporate member of Freie Universität Berlin, Humboldt-Universität zu Berlin, and Berlin Institute of Health (BIH), Institute for Medical Psychology, Luisenstr. 57, Berlin, 10117 Germany; 3 Department of Epidemiology and International Public Health, School of Public Health, Bielefeld University, Bielefeld, Germany; 4 Development, Health and Disease Research Program, University of California, Irvine, CA USA

**Keywords:** Transmission of health inequalities, Stress biology, Turkish immigrants, Pregnancy and birth

## Abstract

**Background:**

Immigrants in Germany exhibit higher levels of social disadvantage when compared to the non-immigrated population. Turkish-origin immigrants constitute an important immigrant group in Germany and show disparities in some health domains that are evident from birth onwards. Several studies have shown the mechanisms by which social disadvantage is biologically embedded to affect health over the lifespan. Relatively little, however, is still known about if and how the maternal social situation is transmitted to the next generation. This study therefore aims to analyse the effects of maternal socioeconomic status and migration status on stress-related maternal-placental-fetal (MPF) biological processes during pregnancy on infant birth and health outcomes.

**Methods:**

This longitudinal cohort study of *N* = 144 child-mother dyads is located at two study sites in Germany and includes pregnant women of Turkish origin living in Germany as well as pregnant German women. During pregnancy, MPF stress biology markers from maternal blood and saliva samples, maternal socio-economic and migration-related information, medical risk variables and psychological well-being are assessed. After birth, infant anthropometric measures and developmental outcomes are assessed. The same measures will be assessed in and compared to Turkish pregnant women based on a collaboration with BABIP study in Istanbul.

**Discussion:**

This is the first study on intergenerational transmission of health disparities in Germany with a focus on women of Turkish-origin. The study faces similar risks of bias as other birth cohorts do. The study has implemented various measures, e.g. culturally sensitive recruitment strategies, attempt to recruit and follow-up as many pregnant women as possible independent of their social or cultural background. Nevertheless, the response rate among lower-educated families is lower. The possibility to compare results with a cohort from Turkey is a strength of this study. However, starting at different times and with slightly different recruitment strategies and designs may result in cohort effects and may affect comparability of the sub-cohorts.

**Trial registration:**

N.A. (Observational study, no clinical trial, no interventions on human participants).

## Background

Immigration to Germany is at its highest number in nearly two decades, and diversity of the population in Germany is increasing. 38% of children under the age of five in Germany belong to families with migrant background [1]. Immigrants, particularly those from low-income to high-income countries, are significantly more likely to experience social disadvantage when compared to the non-immigrated population that may – in part – underlie the observed health differences between host and immigrant populations. Our focus on Turkish-origin individuals in the current study is guided by the consideration that this segment of the German population *a*) constitutes one of the largest migration groups in Germany, defined by country of origin [2]; *b*) is especially socially disadvantaged [3, 4]; *c*) displays health disparities relative to the native German and other minority populations in the country [5–17]; and *d*) exhibits a decline in some aspects of health as a function of duration of residence in Germany and across generations [10, 18].

**Fig. 1:**
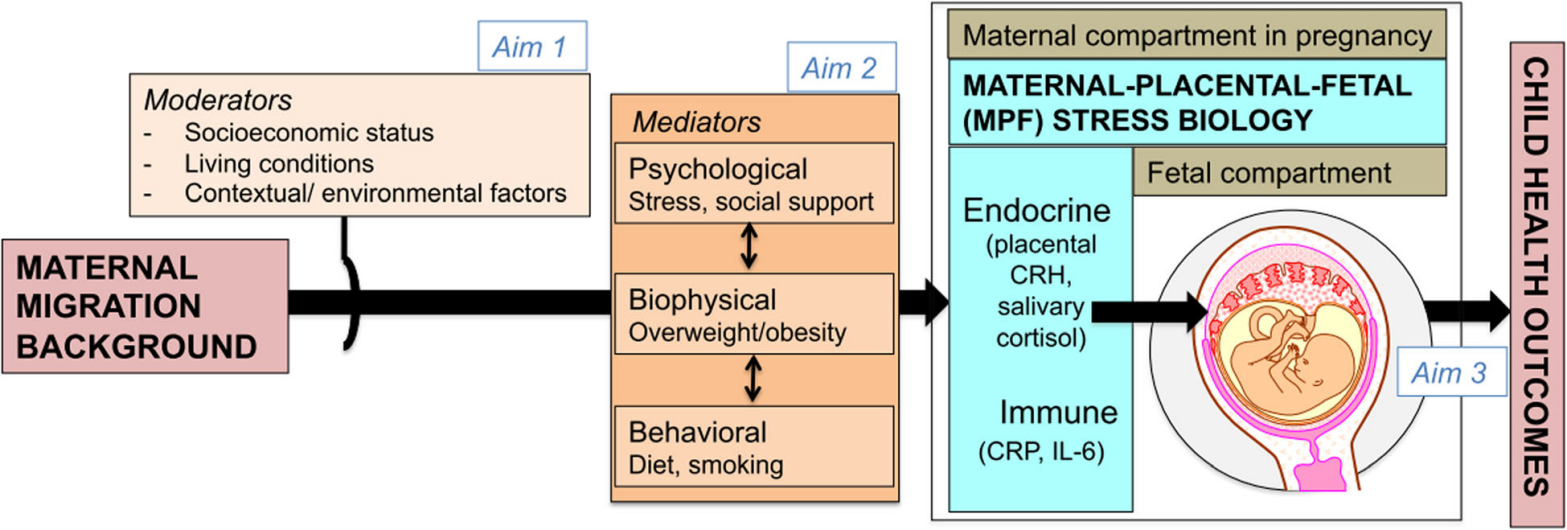
Conceptual framework and study aims

A substantial body of research has uncovered the cascading pathways and underlying mechanisms by which social disadvantage gets biologically embedded “under the skin” to impact health and disease risk across the life span [19]. However, this body of work treats the emergence of health inequalities as a process that operates within the individual life span. Relatively little research has addressed the question of whether and how the effects of social disadvantage may potentially be transmitted across generations [20–22]. Origins of population health disparities may, in part, trace back to the intergenerational effects of maternal migration-related experiences and social disadvantage on her child’s development during the critical period of intrauterine life [23, 24], and we advance the hypothesis that context- and time-inappropriate levels of the resultant stress biology exposures (maternal-placental-fetal endocrine and immune/inflammatory state) during embryonic and fetal life may “program” the structural and functional integrity of cells, tissues and organ systems in a manner that impacts subsequent health and disease risk-related outcomes. As an essential first step towards addressing this hypothesis, we seek to elucidate, in a population-based cohort of pregnant women, in particular of Turkish-origin, and native German mother-child dyads at two study sites located in Bielefeld (BaBi-Stress) and Berlin (BaBeK), the effects of maternal migration status and social disadvantage on stress-related maternal-placental-fetal (MPF) biological processes across gestation. We suggest that stress-related MPF endocrine and immune processes in gestation constitute an attractive underlying biological candidate mechanism for transmitting the effects of different maternal exposure to the fetus for the following reasons: First, these measures are among the most reliable, objective indicators of human fetal exposure to in utero stress (see Fig. 1). A number of animal and human studies, including our work, suggest that perturbations in MPF endocrine and immune/inflammatory stress biology are produced by a broad array of intrauterine conditions [23]. Second, epidemiological, clinical, cellular and molecular evidence converge to suggest that stress-related endocrine and immune processes may serve as a key common physiological pathway (sensors, transducers and effectors) to mediate the effects of intrauterine perturbations on the fetal targets that regulate physiology [25, 26]. Therefore, we assess endocrine (placental corticotrophin-releasing hormone (pCRH), cortisol, and inflammatory (interleukin (IL)-6; C-reactive protein (CRP)) measures of MPF biology serially over the course of gestation as key markers of exposure to adverse intrauterine conditions.

This is the first study on fetal programming of health disparities with a focus on Turkish-origin women in Germany, including a comparison cohort of German women in Germany and the possibility to compare the measures with those assessed in a cohort of Turkish pregnant women in Turkey (based on a collaboration with BABIP study) [27]. The significance and impact of this study derives from the importance of achieving a better understanding of underlying mechanisms that alter disease vulnerability in minority and disadvantaged populations. Aims of the study are:

- To test the hypothesis that a) migration background predicts MPF stress biology during pregnancy, and b) the association between migration background and MPF stress biology during pregnancy is moderated by other social determinants of health.
- To quantify relative contribution of maternal psychological (stress, social support), behavioral (diet, physical activity, substance use) and biophysical (maternal pre-pregnancy BMI and weight gain) states in the overall association of maternal migration background and socioeconomic status (SES) with maternal stress biology.
- To quantify the association of maternal migration background and social disadvantage-related MPF stress biology measures on child birth and health outcomes.

## Methods and design

This study is a multi-centric cohort study to investigate the effects of maternal migration status and SES on stress-related MPF biological processes across gestation and on subsequent infant birth and health outcomes. The study is located at two study sites: BaBi-Stress study in Bielefeld (Germany) and BaBeK study in Berlin (Germany). The cohort will include *N* = 144 pregnant women with their children (child-mother dyads) with serial measures across gestation and birth through 1 month postpartum.

BaBi-Stress in Bielefeld started in 2015 as an add-on study to the larger BaBi birth cohort study [28]. BaBeK was established by the BaBi-Stress study team in Berlin in 2016. Recruitment was established at these two study sites in order to include sufficient numbers of women of Turkish-origin within the study period. The study population consisting of these two sub-cohorts will enable us to disentangle the impact of ethnic and migration specific influences on maternal stress and child health outcomes.

At both study sites, pregnant women are being recruited through gynecologists and midwifes as well as ads and flyers. All study materials including questionnaires are available in German and Turkish language. If necessary, study visits and interviews are conducted by Turkish speaking study nurses. Inclusion criteria were women ≥18 years, singleton intrauterine pregnancy, <= 24 weeks pregnant, German or Turkish speaking, legal residence in Germany, and no chronic maternal diseases or severe pregnancy complications.

Exclusion criteria were younger than 18 years of age, twin or multiple pregnancy, > 24 weeks pregnant, not German or Turkish speaking, and resident of countries other than Germany. German or Turkish origin/ethnicity were not an inclusion criteria because different concepts of migrant background/ origin/ ethnicity exist. We conducted detailed assessments of family background and immigration history after inclusion into the study. Specifically, information on country of birth of the participating pregnant woman and of her parents (mother and father) enables us to describe the migration history of participants more precisely than using measures like nationality/ citizenship or self-assigned origin.

**Fig. 2:**
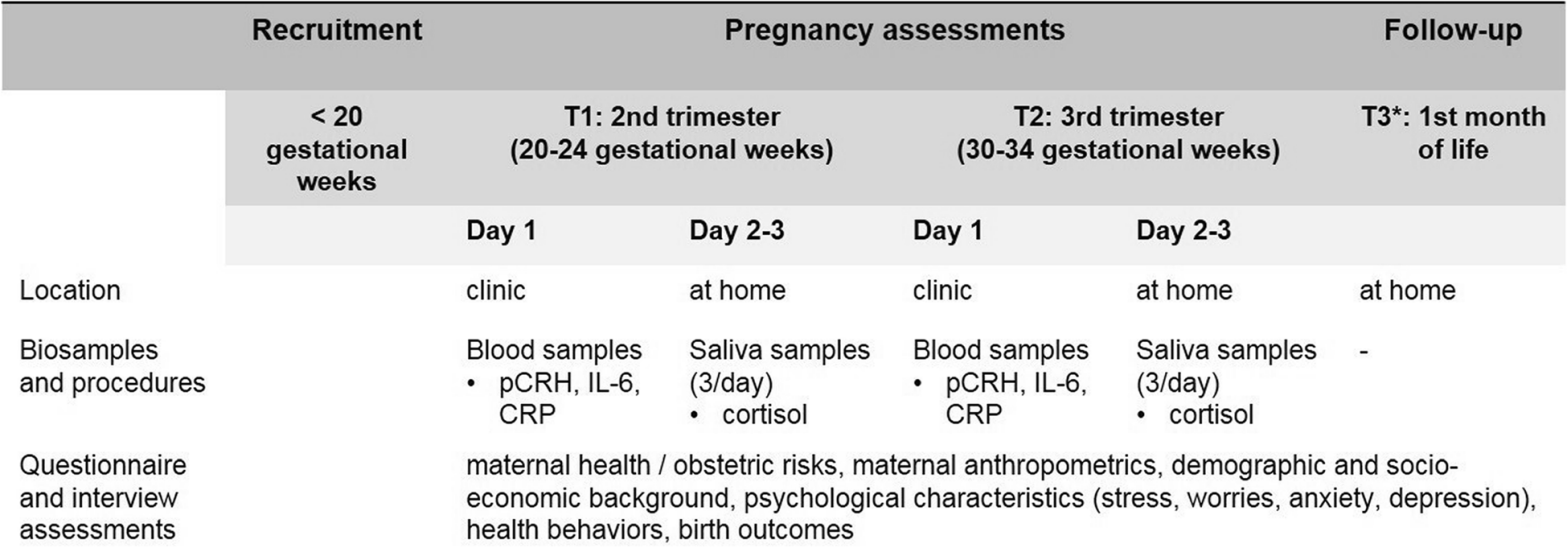
Data collection and follow-up

The study design comprises two study visits in Bielefeld and three study visits in Berlin (see Fig. 2), at the second (T1) and third trimester (T2) during pregnancy and 1 month postpartum (T3, in Berlin only).

We are assessing maternal socio-economic and migration-related information, psychological stress and well-being, nutrition, medical risk variables, and MPF stress biology markers from maternal blood and saliva samples. In Berlin (BaBeK study), additionally infant anthropometric measures and developmental outcomes are collected during infancy.

Table 1 gives an overview of all outcome measures regarding MPF biology of the participants and the physical and cognitive development of their newborns.

**Table 1:** List of outcome measures

Measure	Measured by	Objectives
**MPF biology**		
- Placental corticotropin-releasing hormone (CRH) - Interleukin (IL)-6, - C-reactive protein (CRP) - Cortisol	- Blood and saliva sample during pregnancy at T1 and T2	To measure and analyse differences in endocrine and immune stress markers during pregnancy
**Birth and developmental outcomes**		
- Birth weight & length - Birth outcomes (delivery, complications) - Length of gestation	- Abstraction of medical data from obstetric record at birth	
- Current weight & length - Chronic disease - Physical disabilities - Mental disabilities - Neurodevelopmental disorders	- Abstraction of medical data from obstetric and child examination records at birth and during routine child health examinations (“U-Untersuchungen”) in first month of life	To measure birth and infant anthropometric and developmental outcomes and their association with mothers MPF biology during pregnancy
- Reported developmental physical impairments - Reported neurodevelopmental disorders	- Reported by mother at T3	

An overview of questionnaires used, the time points of assessment and references is given in Table 2.

**Table 2:** Questionnaires used at time points T1, T2, and T3

Questionnaire	T1	T2	T3ª
Cohen’s Perceived Stress Scale(PSS) [29]	X	X	X
Center for Epidemiological Studies – Depression Scale (CES-D) [30]	X	X	X
Spielberger State Trait Anxiety Inventory (STAI) [31]	X	X	X
Cambridge Worry Scale (CWS) [32]	X	X	
Life Experiences Survey (LES) [33]	X	X	
Childhood Trauma Questionnaire (CTQ) [34]		X	
Big Five Inventory (BFI-10) [35]	X		
Partnership questionnaire (PFB) [36], Relationship Assessment Scale (RAS) [37], Dyadic Adjustment Scale (DAS-12) [38]		X	X
Frankfurt Acculturation Scale (FRAKK) [5]	X		
Multidimensional Acculturative Stress Scale for Turkish-origin immigrants (MASI) [39]	X		
Single items about perceived discrimination, language use, intercultural contacts and religion from socioeconomic panel study (SOEP) [40]	X	X	
Resources and self-management skills (FERUS) [41]	X		X
Social Activities (SASS) [42]	X	X	
Edinburgh Postnatal Depression Scale (EPDS) [43]			X
Postpartum Bonding Questionnaire (PBQ) [44]			X
DEGS-food intake questionnaire [45]		X	

^ª^T3 only in BaBeK

### Measures of MPF stress biology

In all two sub-cohorts, blood and saliva samples to assess concentrations of MPF endocrine and immune stress markers are collected at the same two time windows (T1, 20–24 weeks, and T2, 30–34 weeks gestation) during pregnancy (see Fig. 2). The two specific times were selected because the change in endocrine and immune parameters from early to late gestation is critical in characterizing the MPF endocrine and immune stress trajectory with respect to its impact on birth and child developmental outcomes [46, 47].

In Bielefeld and Berlin, maternal blood samples were drawn by the attending prenatal care medical personnel. Women were asked to collect 3 saliva samples per-day on two consecutive days for each pregnancy visit, at awakening, 30-min after awakening, and at 8 pm, which will enable assessment of the diurnal variation of cortisol secretion. Saliva was collected using Salivettes (Sarstedt). The MEMS® (Medication Event Monitoring Systems, AARDEX) system (electronic bottle caps) was used to time and date stamp instances of saliva collection at home. Women were asked to fill out a short questionnaire about their sleep and daily activities and other extraordinary events (medications, gum bleeding etc.) during the saliva collection days. Salivettes were stored in the freezer until they were picked up by a study team member. From the blood samples, CRH and immune biomarkers (CRP, IL-6) are being analyzed, and from the saliva samples cortisol is being analyzed using enzyme-linked immunosorbent assays (ELISAs).

### Psychological, demographic and health measures

A number of psychological constructs are being assessed by validated questionnaires, including: perceived and pregnancy-specific stress, worries, critical life events, anxiety, depression, post-partum depression, relationship satisfaction, acculturation, acculturative stress, and perceived discrimination. Information on socioeconomic and migration-related factors are assessed during the interview at T2, including household income, maternal and partner educational and job attainment, migration history of the participant, her parents and her partner (country of origin, residence status, length of stay, language competencies). Health-related information that is also assessed during the interview includes past and current non-communicable and infectious diseases, psychological disorders, health-related behaviors (physical activities, doctor visits, alcohol consumption, smoking, nutrition), and pregnancy history (number of past pregnancies, complications, risk factors). In the BaBeK study (Berlin), information on birth outcomes (incl. birth weight and height, gestational length, medical risk factors) was extracted from medical records (the so called “Mutterpass” and “U-Heft” documents that contain all medical information during pregnancy and from birth, gathered during medical examinations) 1 month postpartum. In Bielefeld, information on birth outcomes was assessed by a follow-up questionnaire that was sent to the mothers during the newborn’s first year of life as well as data extracted from the BaBi birth cohort, if women participated in both studies.

### Participants

The first pregnant women were enrolled in the study in 2016 in Bielefeld and in 2017 in Berlin. Recruitment has been completed in Bielefeld in April 2017 (*n* = 84) and in Berlin in summer 2018 (*n* = 60). Numbers of enrolled women by country of origin are shown in Table 3. Data analysis of MPF biology has started with the data of the Bielefeld and Berlin study sites and will be finished including the comparisons with the data from BABIP cohort in Istanbul in 2020.

**Table 3:** Number of participants (pregnant women) based on country of birth of the women and their parents

	BaBi-stress and BaBeK-study		
	Participant	Participant’s mother	Participant’s father
Total	144	144	144
Germany	114 (79.17%)	92 (63.89%)	87 (60.42%)
Turkey	19 (13.19%)	30 (20.83%)	33 (22.92%)
Other	8 (5.56%)	16 (11.11%)	20 (13.89%)
Not known	3 (2.08%)	6 (4.17%)	4 (2.78%)

### Statistical power and analysis

We based the statistical power analyses on simulation models we developed for this purpose that incorporate conservative estimates of published effect sizes from our previous studies on group comparisons related to MPF biology. After verifying that the simulation reproduced the assumed pattern of relationships, we used SAS PROC MIXED to perform the planned analyses for each of the specific aims on the simulated data. The standard errors from these analyses were used to generate power estimates for a sample size of *n* = 50 per group (high vs. low stress exposure) and α = .05 (two-tailed tests). Based on these estimates, the minimum effects for differences in measures of MPF biology (CRP, CRH, IL-6, cortisol) by maternal background detectable with 80, 90 and 95% power were 0.12, 0.16 and 0.20, respectively (which correspond to effects described as “small” to “medium” by Cohen [48] reflecting the lower end of our expected effect sizes). Based on standard deviations of data on MPF biological parameters in our previous cohorts of pregnant women, this corresponds to mean differences between groups of 0.626, 0.822, 1.027 mg/dL for CRP; 19.855, 26.474, 33.092 pg/mL for CRH; 0.133, 0.178, 0,222 pg/mL for IL-6; and 0.033, 0.0438, 0.0548 μg/dL for salivary cortisol, respectively. Thus, the proposed sample size will provide adequate statistical power to test our hypotheses concerning within-person main effects, the moderating effects of between-person factors, and the interaction of the two.

In the comparison cohort of Turkish pregnant women in Istanbul (BABIP study) measures of more than 50 women will be available [27], thereby increasing power and variance of the study sample.

Relationships between maternal immigration background and MPF biology will be examined using regression models. For outcomes with repeated measurements (e.g., MPF biology assessed at two time points), mixed-effects regression models will be used. When models include correlated predictors, measures of variance inflation will be applied to assess potential problems of multicollinearity, and the appropriate scaling factors will be employed.

In line with the aims of the study, data analyses are currently ongoing. Results will be published in international scientific journals. First results have been presented at international conferences [49].

A qualitative data pilot study has been conducted to better understand the determinants of stress during pregnancy among women of Turkish origin. This qualitative study on the “Influence of the Turkish migrant background on the subjective feeling of stress in pregnancy” has identified migration-specific, psychological stress categories in pregnant Turkish-origin women, such as pressure from the family and social environment to start a family, conflicts and disputes within the family, lack of support from the partner, financial- and housing-related concerns, pressure to get a boy as a son and heir, pressure to be a perfect mother, and concerns about the cultural education of the child (to teach the child the language, values and beliefs of the culture of the country of origin). Results of this pilot study have been presented at a conference [50]. In future studies, these migration-specific, psychological stress-related constructs should be taken into account in the development of culturally-sensitive instruments for the assessment of maternal stress during pregnancy [50].

## Discussion

This is the first study on fetal programming of health disparities with a focus on immigrant women, in particular on women of Turkish-origin in Germany that includes comparison cohorts of German women in Germany and the opportunity to compare the measures with a cohort of Turkish women in Turkey. The results contribute to a better understanding of the underlying mechanisms that affect disease vulnerability in minority and disadvantaged populations and the transmission of risks across generations.

**Fig. 3:**
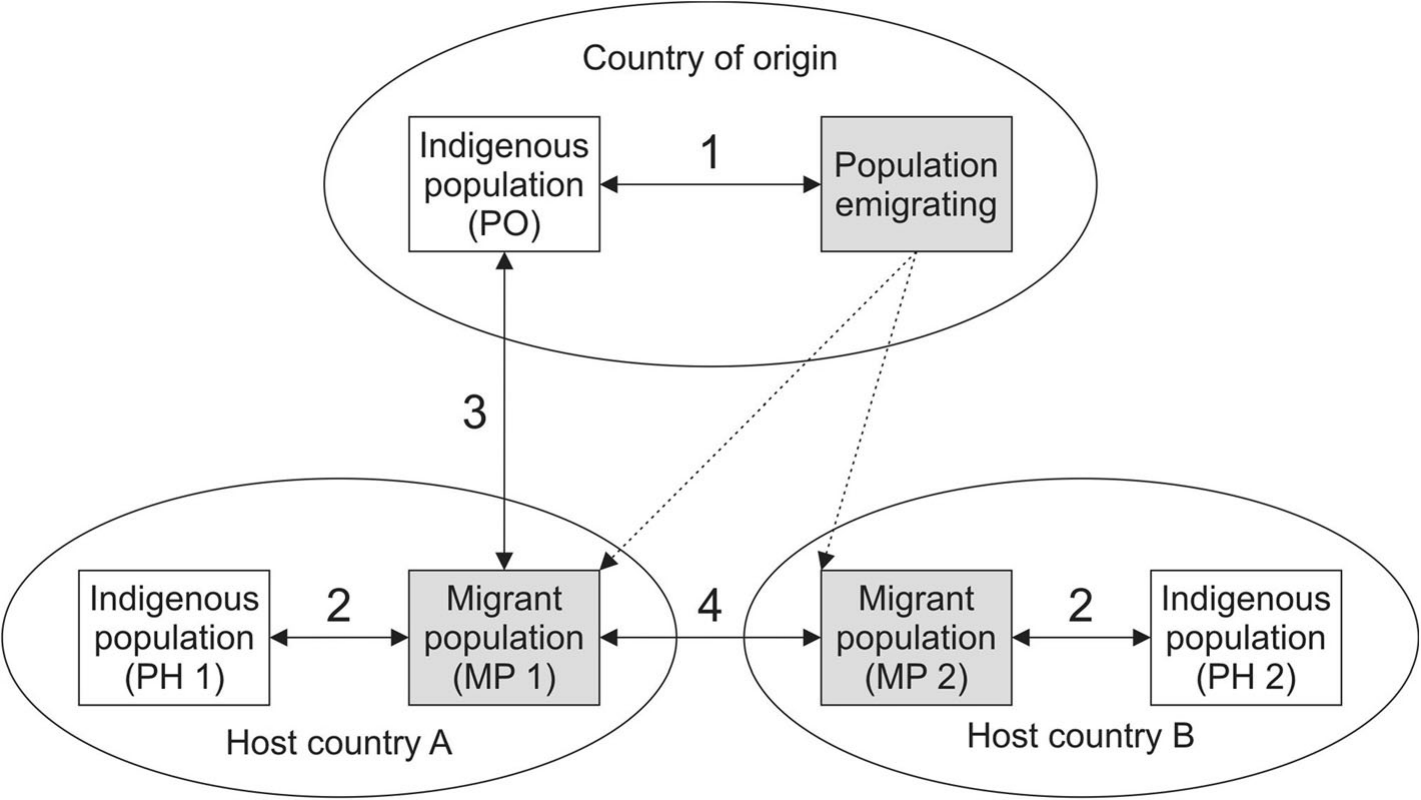
Possibilities (1–4) to compare health of migrants with health of autochthonous (indigenous) populations [51]

An ideal cohort study researching migrant health should not only include immigrants and a non-immigrated population in the new home country (“host country”), but also participants in the country of origin [51]. As suggested by Spallek et al. [51] this study is to our knowledge the first cohort study in Germany that allows to compare the situation of Turkish migrant women and their offspring living in two different cities in Germany with non-migrated women in Germany (Comparison 2 in Fig. 3) and with non-migrated women in Turkey (Comparison 3). For the future, it would be desirable also to include additional sub-cohorts of Turkish migrant women in other host countries to make the suggested comparison 4 also possible.

The study faces comparable risks of bias as other population-based birth cohorts. Bias due to self-selection of participants cannot be ruled out, as the study relies on voluntary participation. To reduce this bias and to recruit and follow-up as many pregnant women as possible independent from their social or cultural background, the study implemented various measures such as culturally sensitive recruitment, bilingual study materials and bilingual study nurses and public relations. Nevertheless, the response rate among families with a migrant background was lower than among those without a migrant background in Bielefeld and Berlin. In addition, in Bielefeld the response rate was lower among lower-educated families as compared to higher-educated families. The study is located at two study sites. The possibility to compare measures with a comparison cohort from Turkey is a strength of this study. However, starting at different times and with slightly different recruitment and design might result in cohort effects between the different cohorts and decrease comparability. Nevertheless, comparing measures of women from different environments is increasing the variance of the sample, allowing more sophisticated analyses, increasing the generalizability, and finally allowing comparison with the situation of Turkish women in Turkey.

## Data Availability

The datasets analyzed during the current study are not publicly available due to data protection restrictions as individual privacy could be compromised due to the small number of participants and the high specifity of the study population. Data and questionnaires will be made accessible to researchers by the corresponding author on reasonable request and in line with data protection regulations.

## Abbreviations

BMI: Body mass index
CRH: Corticotrophin-releasing hormone
CRP: C-reactive protein
ELISAs: Enzyme-linked immunosorbent assays
IL-6: Interleukin-6
MEMS®: Medication Event Monitoring Systems
MPF: Maternal-placental-fetal
pCRH: Placental corticotrophin-releasing hormone
SES: Socioeconomic status
